# SGO1 Maintains Bovine Meiotic and Mitotic Centromeric Cohesions of Sister Chromatids and Directly Affects Embryo Development

**DOI:** 10.1371/journal.pone.0073636

**Published:** 2013-09-03

**Authors:** Feng-Xia Yin, Guang-Peng Li, Chun-Ling Bai, Yang Liu, Zhu-Ying Wei, Cheng-Guang Liang, Thomas D. Bunch, Lin-Sen Zan

**Affiliations:** 1 The Key Laboratory of National Education Ministry for Mammalian Reproductive Biology and Biotechnology, Inner Mongolia University, Hohhot, China; 2 College of Animal Science and Technology, Northwest A&F University, Yangling, Shaanxi, China; 3 Department of Animal, Dairy and Veterinary Sciences, Utah State University, Logan, Utah, United States of America; Institute of Zoology, Chinese Academy of Sciences, China

## Abstract

Shugoshin (SGO) is a critical factor that enforces cohesion from segregation of paired sister chromatids during mitosis and meiosis. It has been studied mainly in invertebrates. Knowledge of SGO(s) in a mammalian system has only been reported in the mouse and Hela cells. In this study, the functions of SGO1 in bovine oocytes during meiotic maturation, early embryonic development and somatic cell mitosis were investigated. The results showed that *SGO1* was expressed from germinal vesicle (GV) to the metaphase II stage. SGO1 accumulated on condensed and scattered chromosomes from pre-metaphase I to metaphase II. The over-expression of SGO1 did not interfere with the process of homologous chromosome separation, although once separated they were unable to move to the opposing spindle poles. This often resulted in the formation of oocytes with 60 replicated chromosomes. Depletion of *SGO1* in GV oocytes affected chromosomal separation resulting in abnormal chromosome alignment at a significantly higher proportion than in control oocytes. Knockdown of *SGO1* expression significantly decreased the embryonic developmental rate and quality. To further confirm the function(s) of SGO1 during mitosis, bovine embryonic fibroblast cells were transfected with *SGO1* siRNAs. *SGO1* depletion induced the premature dissociation of chromosomal cohesion at the centromere and along the chromosome arm giving rise to abnormal appearing mitotic patterns. The results of this study infer that SGO1 is involved in the centromeric cohesion of sister chromatids and chromosomal movement towards the spindle poles. Depletion of *SGO1* causes arrestment of cell division in meiosis and mitosis.

## Introduction

Meiosis is a unique genetic phenomenon in which DNA replicates once followed by two sequential cell divisions. During the first meiotic division (meiosis I), the homologous chromosomes are paired and crossing over occurs with the formation of a chiasmata. Each pair of sister chromatids remain tightly linked until all chromosomes are aligned at the equatorial plate and attached to the meiotic spindle at metaphase via their kinetochores [1]. Sister chromatids are held together along the chromosomal arms by a ring-like multi-subunit cohesin protein complex [2], [3]. The sister chromatid cohesion at the centromere is retained until meiosis II, when sister chromatids segregate by the mediation of separase, a complex catalyzing cohesin dissociation [2]. Protection of centromeric cohesin from premature dissociation is thereby controlled by shugoshin (SGO) during mitosis [4]–[6], and meiosis [7]–[10].

Two members of the shugoshin protein family have been reported. Shugoshin 1 (SGO1) exists in fission yeast [11], budding yeast [12], [13], fruit flies [14], *Xenopus* laevis [15] and Hela cells [5], [16], [17]. SGO2 has been reported to be in fission yeast [18], [19] and vertebrates [2], [3]. In vertebrate mitotic cells, the majority of the cohesin complex dissociates from the chromosomal arms when a cell enters prophase [20], [21]. The depletion of human *SGO1* (*hSGO1*) results in the removal of cohesins even as centromeres pass through the prophase pathway [5], [17], [22]. The depletion leads to the precocious separation of sister chromatids before metaphase. In an *in vitro* system, purified hSGO1 dephosphorylates the SA2 subunit of the cohesin complex from the originally phosphorylated state by the Polo-like Kinase 1(PLK1). Okadaic acid treatment will diminish the reaction [20]. In human cells, the knockdown of *SGO2* causes a mild defect in the centromeric protection of cohesin, and results in chromosomal mal-alignment defects [20], [21]. These observations suggest that SGO2 may have dual roles in establishing bipolar attachment in human cells. *Sgo2*-knockout mice develop normally and embryonic fibroblast cells proliferate with no obvious mitotic defects [23].

Mouse SGO1 and SGO2 are ubiquitously expressed in proliferating cells, and SGO2 expression level has been reported to be higher in testis and oocytes [24]. During mouse meiosis I and II, SGO1 and SGO2 localizes around the inner kinetochore region where the cohesin complex forms [24]. Analysis of *Sgo2*-depleted mouse oocytes have shown that precociously separated chromatids were frequently observed at metaphase II, but not during meiosis I. These observations indicate that in oocytes, *Sgo2* depletion abolishes sister centromatid cohesion during anaphase I [24]. It has also been reported that treatment of oocytes with okadaic acid (a phosphatase inhibitor) induces premature separation of sister chromatids during meiosis I [25]. Similar phenomena have been observed in nicotine-exposed bovine oocytes [26], [27].

Defects in the regulation of chromosome separation will result in arrestment of cell division, aneuploidy and tumorigenesis [28]–[31]. Abnormal expression of SGO1 and related factors can lead to chromosomal mis-separation and cellular developmental failure [32]. Yamada et al. reported that haplo insufficiency of *SGO1* caused enhanced chromosomal instability and colon tumorigenesis in the mouse [33]. Compared to normal tissues, the expression level of *hSGO1* is lower in tumor tissues of colorectal cancer patients [34] and higher in tumor tissues of breast carcinoma patients [35]. *SGO1* RNAi has been reported to induce transformed cells into apoptosis [36].

Much of the information obtained about mammalian SGO in meiosis has been obtained from mice. In this study we use the bovine model and investigate the roles of SGO1 during oocyte meiotic maturation and early embryonic development with exogenous over expression and by RNA interference. The role of bovine SGO1 in fibroblast cells during mitosis was also investigated and compared to the results obtained from the meiotic studies.

## Materials and Methods

### Ethics Statement

All studies adhered to procedures consistent with the National Research Council Guide for the Care and Use of Laboratory Animals and were approved by the Institutional Animal Care and Use Committee at Inner Mongolia University.

### Chemicals

Chemicals and medium used in this study were purchased from Sigma Chemical Co. (St. Louis, MO) unless otherwise indicated. Primers were synthesized by Takara Biotechnology Dalian Co. Ltd (Dalian, China). Sequencing assays were performed by Life Technologies Corporation.

### Maturation of Oocytes *in vitro* (IVM)

Maturation of bovine (*Bos taurus L.*) oocytes was performed as previously described [37]. Bovine cumulus oocyte complexes (COCs) were aspirated from 3–8 mm diameter follicles on ovaries that were collected from a local abattoir (Xiyuan, Hohhot, China) with their permission. Only COCs with at least 3 layers of cumulus cells and a compact and homogenous ooplasm were selected for use. The oocyte maturation medium consisted of TCM 199 with Earle salts, L-glutamine, and sodium bicarbonate (Gibco Inc., Grand Island, NY), supplemented with 10% fetal bovine serum (FBS) (HyClone, Logan, UT), 0.01 µg/mL E2(Estradiol), 0.01 IU/mL FSH (Follicle stimulating hormone) and 1 IU/mL LH (Luteinizing hormone). 30–50 oocytes were cultured in 0.5 mL maturation medium per well in 4-well plates.

### Parthenogenetic Activation and *in vitro* Development

Upon maturation, oocytes with a PB1 (the first polar body) were activated by 5 µM ionomycine for 5 min, then treated with 10 µg/mL cycloheximide (CHX) and 5 µg/mL CB(Cytochalasin B) in CR1aa (Charles Rosenkrans 1 amino acid) plus 3% FBS for 5 h at 38.5°C in 5% CO2 in air. Oocytes were then cultured in 40 µL droplets of CR1aa contained 0.3% bovine serum albumin (BSA) for 40 h. Cleaved embryos were transferred to CR1aa supplemented with 4% FBS medium and placed upon a feeder layer of bovine cumulus cells and incubated in 5% CO_2_ in air at 38.5°C for 7 days. One-half of the medium was replaced every two days by fresh CR1aa supplemented with 4% FBS medium.

### SGO1 Plasmid Construction

Total RNA was extracted from 100 GV oocytes using a Fast RNA Micro Kit (Watson) and the first strand cDNA was generated with RT-PCR kit (Takara) using oligo (dT) primers. The following primers were designed according to the sequences in Genbank (accession number: BC133396.1, 09-JUN-2008) and were used to clone the full length of *SGO1* cDNA. Recognition sequences of restriction enzymes Ba*mH* I and N*ot* I (underlined) were added up stream of the forward and reverse primers, respectively.

F: 5′ GGATCC TGGTGTGAGCAGAGCAGCAG 3′

R: 5′ GCGGCCGC TGTACTTGCTGCACATTTTT 3′

The *SGO1* cDNA was inserted into the multiple clone site (MCS) of pCDNA3.1 (+) myc-hisB vector (Invitrogen) driven by a T7 promoter via N*ot* I and Ba*mH* I. The downstream myc tag was used to detect SGO1 signals.

### *In vitro* Synthesis of mRNA

For the *in vitro* transcription reactions, the pCDNA3.1 (+)-*SGO1*- myc-hisB plasmid was linearized by restriction enzyme D*ra* III and purified by SV Gel and PCR Clean-up (promega). A T7 message machine kit (Ambion) was used to produce capped mRNA, which was then purified using the RNA clean kit (TIANGEN). The pCDNA3.1 (+) - myc-hisB plasmid was also linearized and performed to produce *MYC* mRNA as controls. The concentration of *SGO1-MYC/MYC* mRNA was detected by NANODROP 2000c (Thermo Fisher, PIT, USA), and then the mRNA was diluted into a lower concentration (0.3 mg/ml) for localization tracking and to a higher concentration (3.0 mg/ml) for overexpressing the protein. The forward and reverse primers incorporated into the *SGO1* sequences and the plasmid sequences downstream of MYC tag are listed below. They were specifically designed to identify *SGO1-MYC* mRNA upon using RT-PCR.

F: 5′ CAGTCCTAGAGCAGAAGATGGC3′,

R: 5′GTGATGGTGATGATGACCGGTA3′.

### RNA Interference

Based upon information from GenBank, the *SGO1* mRNA interference sequences were designed via software published by Applied Biosystems official website (http://www.ambion.com/techlib/siRNA_finder.html). As shown in Table 1, three pairs of bovine *SGO1* siRNA (small interfering RNA) sequences and one pair of negative control siRNA sequences were synthesized (Takara Company,Dalian, China). The siRNAs used in this study were a mixture of three pairs of *SGO1* siRNA (m-siRNA). Small interfering RNAs were mixed equally before microinjecting into oocytes.

**Table 1:** Details of double-stranded siRNAs.

Target gene	Start sites	siRNA sequence
*SGO1*	668	Sense5′-3′: CUA UAU CUC UCC GUC GUGGtt Antisense5′-3′: CCACGACGGAGAGAUAUAGtt
	954	Sense5′-3′: GCACAGAGGUGCCAAAGAAtt Antisense5′-3′: UUCUUUGGCACCUCUGUGCtt
	1171	Sense5′-3′: GUG AGC ACC UGU GAA UCAAtt Antisense5′-3′: UUG AUU CAC AGG UGC UCACtt
Negative control	No matches	Sense5′-3′: ACU CUA GCU GCG UCU GCU Utt Antisense5′-3′: AAG CAG ACG CAG CUA GAG Utt

### Microinjection of SGO1-MYC mRNA or SGO1 siRNAs

Microinjection of oocytes was performed using an Eppendorf micro injector (Hamburg, Germany) and completed within 30 minutes per treatment group. The injector pipette was replaced after fifty oocytes were injected to avoid possible false negative results. For localization tracking or overexpression, *SGO1-MYC* mRNA solutions at concentrations of 0.3 µg/µl or 3.0 µg/µl were injected into the cytoplasm of GV oocytes. The same amount of MYC was injected as a control. The injected oocytes were arrested at the GV stage in 25 µM Roscovitine for 3 hours before meiotic resumption. 5 pl of *SGO1* m-siRNAs solution were microinjected into the cytoplasm of GV intact oocytes and the injected oocytes were maintained in maturation medium containing 25 µM Roscovitine for 24 h before meiotic maturation. 5 pl of *SGO1* m-siRNAs were also microinjected into the cytoplasm of parthenogenetic pseudo-zygotes which were held in CR1aa. The same amount of non-sense siRNA was injected for the negative control.

### Quantification of SGO1 Expression in Oocytes by Real-time PCR

Analysis of relative gene expression was measured by real-time quantitative PCR using the 2(-Delta Delta C (T)) method [38]. The extraction and reverse transcription of total RNA were performed as described above. The following primers were used for cDNA amplification of *SGO1* with the reference gene being *GAPDH* (glyceraldehyde-3-phosphate dehydrogenase). *SGO1*, forward, CCAGTAGTGACGACAACTCCAGAGA; reverse, CTGGACAGTCAGCACCCTCAAG.

*GAPDH*, forward, GATGGTGAAGGTCGGAGTGAAC; reverse, GTCATTGATGGCGACGATGT. SYBR Premix Ex Taq II kit (Takara) was used for real-time PCR in an ABI prism 7300 Sequence Detection System (Applied biosystems, CA, USA). The steps involved 95°C 30 s, 40 cycles of 95°C 5 s and 60°C 31 s.

### Cell Culture, Synchronization and Transfection

Bovine(*Bos primigenius taurus*) fibroblasts were obtained from the skin of a bovine fetus at day 50, and fibroblasts from passage 3 to passage 5 were used to perform transfections. Embryonic fibroblasts were cultured in DMEM (Dulbeccòs Modified Eagle Medium) supplemented with 10% FBS and 0.2 mM L-glutamine. Cell synchronization was performed as described [39]. Lipofectamine 2000 (Invitrogen) was used for siRNA transfection following the guidelines of the manufacturer. Cells were collected at 5, 7 and 9 h after the second blocking release for chromosome spreading. Chromosome morphology and frequency of occurrence were determined by analyzing more than 100 mitotic cells (chromosome spreads) for each sample in a single experiment.

### Immunofluorescent Microscopy, Chromosome Spreads and Image Analysis

Immunofluorescence was performed as described previously with slight modification [40]. Oocytes were first treated with 1% sodium citrate for 20 minutes to obtain a better resolution of chromosomal configurations, then fixed with 4% paraformaldehyde and permeabilized with 0.2% Triton X-100 in PBS (Phosphate Buffer Solution) overnight at 4°C. After being blocked in PBS plus 3% BSA for 1–2 h at room temperature, oocytes were incubated at 4°C overnight with primary antibody. The c-myc antibody ((R950-25, Life Technologies Corporation, Carlsbad, California) was applied at a dilution of 1∶300. Oocytes were then labeled with FITC conjugated secondary antibody (sc-2010, Santa Cruz Biotechnology Inc., Delaware) diluted 1∶500 for 2 h at room temperature. The nuclei were stained with 10 µg/ml propidium iodide for 5 min. Blastocysts were stained with 5 µg/mL Hoechst 33342 for 10 min. The oocytes and blastocysts were examined with a Zeiss epifluenent microscope (Carl Zeiss Optical, Inc., Chester, VA). Each experiment was repeated three or more times with ≥30 oocytes being examined at each observation. Eighty percent of all samples were quantifiable. Instrument settings were kept constant for each replicate. Images were captured by digital camera with PIXERA Viewfinder Program which greatly aided in the data analyses (Pixera Corporation).

Oocytes were treated with 1% trisodium citrate at room temperature for 10–15 minutes and then fixed quickly by fresh methanol: glacial acetic acid (3∶1) on glass slides for 24 h to obtain chromosome spreads for analysis. Chromosome spreads were stained with 1% Giemsa for 10 min. Cells in mitosis were harvested by trypsinization and treated with 0.075 M KCl for 40 minutes at room temperature, then fixed by fresh methanol: glacial acetic acid (3∶1) for 30 minutes with three repeats. Fixed cell suspensions were dropped onto a glass slide and the slides were dried at room temperature for 24 h. Chromosomes were stained with 1% Giemsa for 20 min and the slides were washed slightly by tap water and dried for microscopic examination. Images were digitally captured using the Nikon Elements Program with a Nikon microscope (KHU, TYO, Japan).

### Statistical Analysis

Data (mean ± SEM) were pooled from at least three replicates per experiment and analyzed by one-way analysis of variance (ANOVA) using origin 8 software. Data were analyzed using the Student t-test with a P-value of <0.05 being considered statistically significant.

### Experimental Design

#### Experiment 1

This experiment was designed to examine the expression levels of *SGO1* during bovine oocyte meiotic maturation. Real-time RT PCR was performed to detect the mRNA level of *SGO1* at 0, 8, 12, 16 and 24 h. The 2 (-Delta Delta C (T)) method was used for data analysis.

#### Experiment 2

This experiment was designed to examine the distribution of SGO1 during oocyte meiotic maturation. COCs were denuded and processed for injection of *SGO1-MYC* mRNA that had been diluted to a lower concentration. Oocytes were collected at 0, 8, 14, 18 and 24 h of culture, which corresponds to GV, germinal vesicle breakdown (GVBD), metaphase I (MI), anaphase/telophase I (AI/TI), and metaphase II (MII) stages. Subcellular distributions of SGO1-MYC were observed with a fluorescence microscope.

#### Experiment 3

This experiment determined whether over-expression of *SGO1* during meiosis I affects chromosome separation. Oocytes at the GV stage were injected with *SGO1-MYC* mRNA at a higher concentration. After meiotic maturation, the oocytes were immuno-stained and analyzed. Concomitantly with the treatment of oocytes, chromosome spreading was performed and analyzed.

#### Experiment 4

This experiment determined whether RNAi of *SGO1* at meiosis I affects chromosome separation. Oocytes at the GV stage were injected with *SGO1*-specific siRNA, followed by treatment of 25 µmol Roscovitine for 24 h to prevent meiotic resumption. After *in vitro* maturation for 24 h, chromosome spreading was performed and the percentage of chromosome anomalies was determined.

#### Experiment 5

This experiment determined whether depletion of *SGO1* in 1-cell embryos affects subsequent development. Zygotes obtained from parthenogenic activation were injected with *SGO1*-specific siRNA, followed by the observation of embryonic development *in vitro*. The cleavage and blastocyst rates were calculated. The cell number of individual blastocysts was determined.

#### Experiment 6

This experiment determined whether RNAi of *SGO1* affects chromosome separation during mitosis in bovine embryonic fibroblasts. Synchronized transfected cells were harvested at different times. Observations were classified according to particular chromosomal configuration patterns.

## Results

### Dynamic Expression of SGO1 in Bovine Oocytes during *in vitro* Maturation

**Figure 1:**
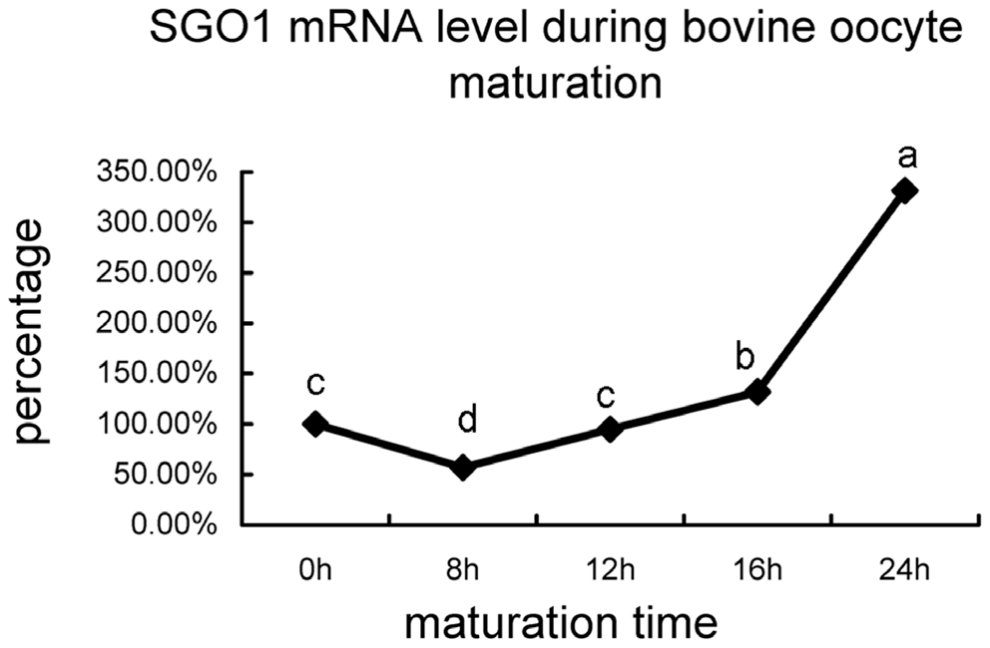
The relative mRNA levels of *SGO1* in a matured bovine oocyte from GV to MII stages. The expression level of *SGO1* at GV stage (0 h) was adjusted to 100%. Different superscripts indicate a statistical difference (P<0.01).

In order to examine the dynamic expression of *SGO1*, oocytes were incubated *in vitro* for 0, 8, 12, 16, and 24 h, which corresponded to GV, germinal vesicle breakdown (GVBD), metaphase I (MI), anaphase I/telophase (AI/TI), and metaphase II (MII) stages, respectively [40]. *SGO1* expression levels were detected by Real-time RT-PCR. *SGO1* mRNA level at GV was normalized to 100%. *SGO1* expression level decreased to 57.0% at GVBD, which was significantly lower than what was observed at GV. The amount of *SGO1* mRNA then increased to 95% at MI and 132.0% at AI/TI. The mRNA amount in MII oocytes was 3.3-fold (331.7%) higher than in GV oocytes (Fig. 1).

### Subcellular Distributions of SGO1 during Bovine Oocyte Maturation

**Figure 2:**
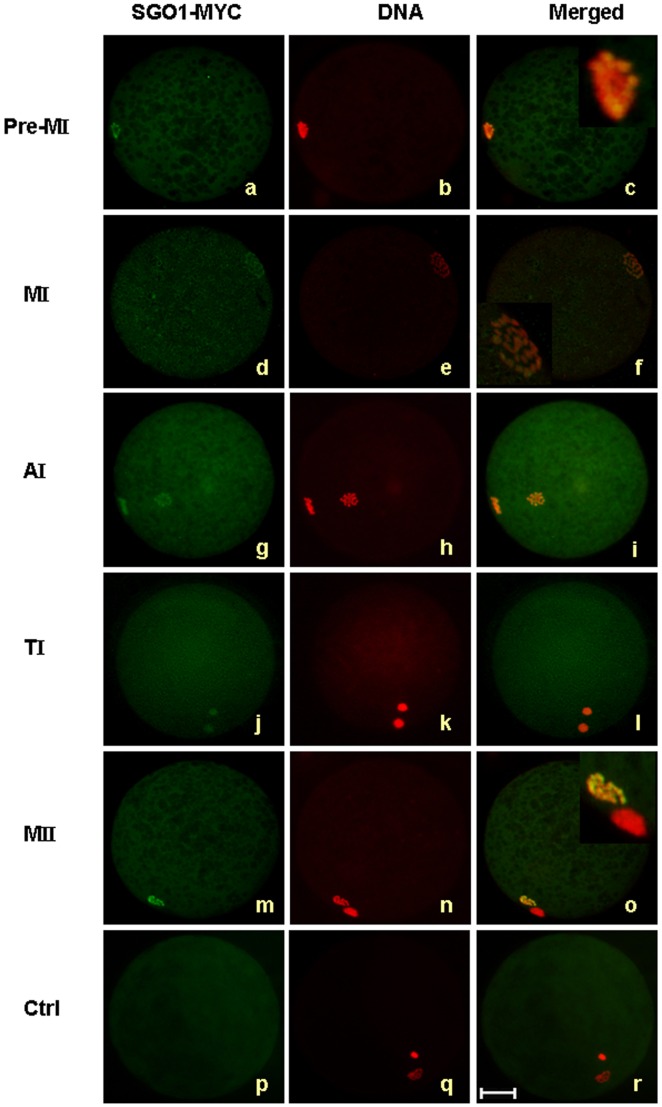
The localization of SGO1-MYC during bovine oocyte maturation. Oocytes matured at different stages were stained with c-myc antibody and PI. Green, SGO1-MYC; red, DNA; yellow, overlapping of red and green. pre-MI, pre-metaphase I; MI, metaphase I; AI, anaphase I; TI, telophase I; MII, metaphase II. Bar = 50 µm; original magnification×400.

Because of the unavailability of an endogenous specific antibody for bovine SGO1, we resorted to using an exogenous delivered *SGO1*. MYC tagged *SGO1* mRNA acquired via *in vitro* transcription was injected into the cytoplasm of GV oocytes, followed by incubation for 0, 8, 14, 18 and 24 h. The oocytes were then denuded for immuno-fluorescent staining with anti-myc antibody. The results showed that at pre-MI stage, SGO1 exhibited a distribution pattern along condensed chromosomes with greater enrichment in some areas (Fig 2 a, b, c). As the oocytes progressed to MI, the chromosomes aligned at the equatorial plate. At this stage of development, SGO1 was observed associated with every dispersed chromosome (Fig 2 d, e, f). At AI, homologous chromosomes were segregated from each other. One set of chromosomes migrated to the periphery and began to condense, which was accompanied with the co-localization of SGO1. The other set of chromosomes presented a “metaphase-like” chromosome spread and SGO1 was concentrated on the outer rim of the chromosomes appearing like a loop. When oocytes developed to TI, SGO1 was then co-localized with hyper-condensed chromosomal masses (Fig 2 j, k, l). In MII oocytes, SGO1 was distributed only at the nucleus, being concentrated in some spots (the enlarged figure) and not detected in the polar bodies (Fig 2 m, n, o). After microinjection of MYC mRNA, MYC displayed no specific localization in bovine oocytes. (Fig 2 p, q, r).

### Over-expression of Exogenously Delivered SGO1 Inhibits Chromosomal Polarization during Oocyte Meiosis

**Figure 3:**
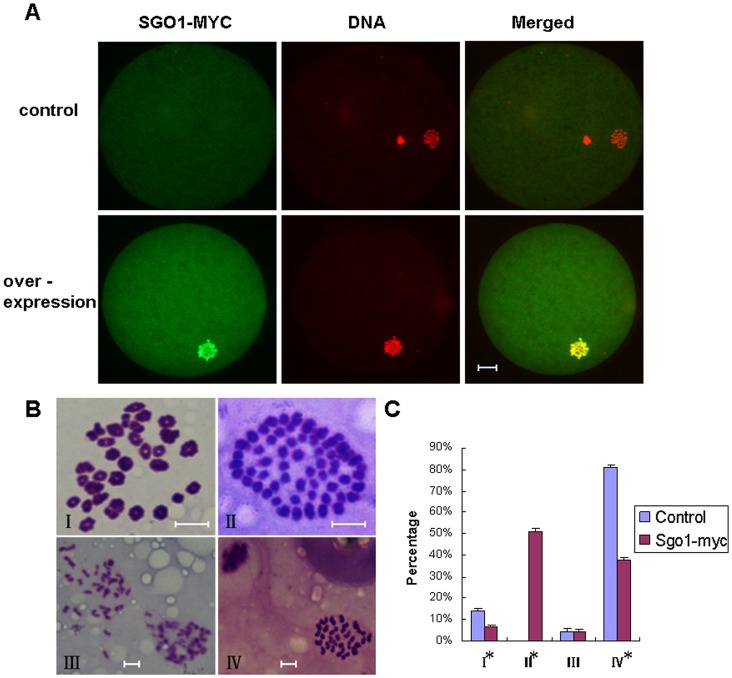
Over-expression of exogenously delivered *SGO1* in bovine oocytes during meiosis inhibited chromosome polarization. A) Images of oocytes without PB1 after *SGO1* over-expression. Oocytes were stained with c-myc antibody and PI. Green, SGO1-MYC; red, DNA; yellow, overlapping of red and green. Bar = 20 µm; original magnification×400.B) Representative chromosome configurations are shown. I, metaphase I; II, 60 chromosomes; III, anaphase I; IV, metaphase II. Bar = 5 µm; original magnification×1000. C) Corresponding figure presentation as in B. The error bar is expressed as mean ± SEM.* indicate a statistical difference (P<0.01).

To investigate the potential role of SGO1 in chromosome separation during oocyte meiosis, GV oocytes were microinjected with 3.6 µg/µl *SGO1-MYC* mRNA followed by incubation in the presence of 25 µM Roscovitine for 3 h to prevent meiotic resumption. The results showed a significant decrease in maturation rate (37.9%) in the treatment group as compared to the control (81.0%). The majority of treated oocytes did not extrude a PB1 (the first polar body). Upon further analysis of these oocytes without a PB1, the chromosomes aligned at the equatorial plate with irregular configurations (Fig.3A). More than 50% of the oocytes without PB1 contained 60 replicated chromosomes with *SGO1* over expression (Fig.3B-II), whereas such phenomenon was not observed in the control. There were no observable differences between the treated and control groups for oocytes arrested at MI or AI (Fig.3B-I). The homologous chromosomes separated from each other after the exogenous overexpression of *SGO1,* but the separated chromosomes were not able to migrate to opposite spindle poles. The percentages of oocytes were calculated according to the chromosomal configuration (Fig.3C).

### Depletion of SGO1 at GV Stage Affects Chromosome Separation

**Figure 4:**
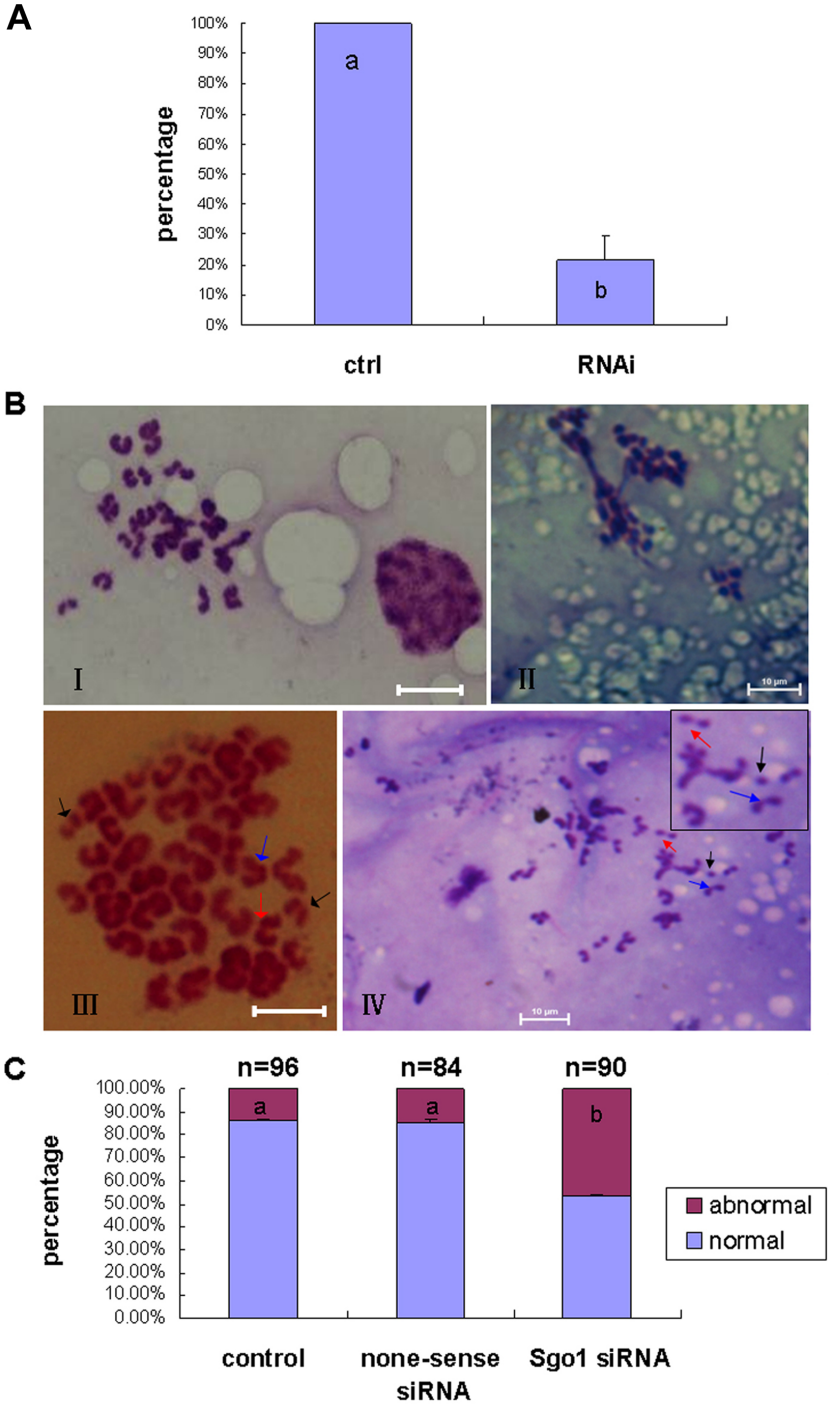
Depletion of bovine *SGO1* in GV oocytes affected chromosome separation. A) *SGO1* mRNA level after RNAi. Different superscripts indicate a statistical difference (P<0.01). The error bar is expressed as mean ± SEM. B) Chromosome spread after *SGO1* RNAi. I: normal metaphase II chromosomes; II, multi-polar chromosome; III and IV, abnormal chromosomal separation, bar = 10 µm; original magnification×1000. C) The percentage of abnormal chromosomes (III and IV) after *SGO1*-specific interference. Different superscripts indicate a statistical difference (P<0.05). The error bar is expressed as mean ± SEM.

To further investigate the function of SGO1 during oocyte meiosis, siRNA oligonucleotides were used to disrupt the expression of *SGO1*. GV oocytes were injected with 20 pM siRNA against *SGO1* and then treated with 25 µM Roscovitine for 24 h to prevent meiotic resumption followed by incubation for 24 h for meiotic maturation. The results indicated that the amount of *SGO1* mRNA was largely reduced after RNAi treatment (Fig. 4A). The oocyte maturation rate significantly decreased after SGO1 depletion (44.6% vs 80.4% in control) (Table 2). Except for rarely seen multi-polar chromosomal masses in all the three groups, the depletion of SGO1 resulted in a higher proportion of oocytes (46.7%) with chromosomal abnormalities as compare to the non-injection control (13.5%) and in the non-sense siRNA injection group (14.4%)(Fig. 4B, C). Typically observed chromosome asynchronous separations consisted of: normal sister chromatids combined at the centromere (blue arrow in Fig.4B); loosened centromeric cohesion in chromatid pairs (red arrow in Fig.4B); and single separated chromatids (black arrow in Fig.4B). The depletion of SGO1 resulted in premature chromatid separation.

**Table 2:** Effects of *SGO1* siRNA on *in vitro* maturation of bovine oocytes.

Treatments	No. IVM oocytes	No. oocytes with PB1 (%)
Control	93	75 (80.4)^a^
*SGO1* siRNA injection	111	49 (44.6)^b^
None-sense siRNA injection	81	63 (77.8)^a^

Within a column, values with different superscripts differ significantly, p<0.01.

### Depletion of SGO1 Decreases Embryonic Development and Embryonic Quality

**Figure 5:**
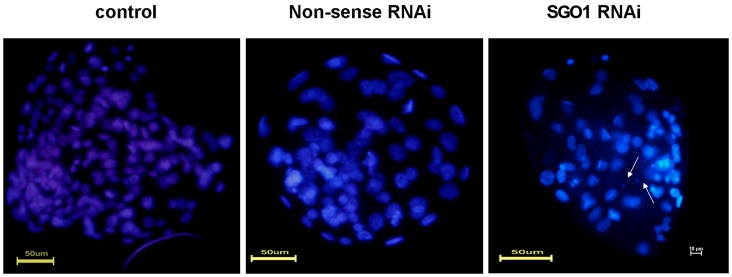
Assessment of cell number of the resulted bovine blastocysts derived from *SGO1*-RNAi embryos. The arrows show fragmented nuclei. Stained with Hoechst 33342. Bar = 50 µm; original magnification×400.

To examine whether SGO1 affected embryonic development, *SGO1* siRNAs were microinjected into the cytoplasm of parthenogenetic pseudo-zygotes. The injected embryos were incubated as previously described [27]. The results indicated that there were no statistical differences between the embryonic cleavage rate in the experimental group and the non-injected and non-sense siRNA injected groups. The occurence of blastocyst development, however, significantly decreased in *SGO1* RNAi embryos (9.4%) when compared to the non-sense injected (16.2%) and the non-injected groups (19.2%) (Table 3). Cell number within blastocysts was significantly lower in the *SGO1* RNAi group (average 65.0 cells) when compared to non-sense group (90.0 cells) and the non-injected groups (105.0 cells) (Fig.5). *SGO1*-specific RNAi dramatically altered embryonic development and blastocyst quality. *SGO1* siRNA treatment induced various micronuclei formations within affected blastomeres.

**Table 3:** Knockdown of *SGO1* by siRNA decreased embryonic development and quality.

Treatments	No. pseudo-zygotes	Cleavage (%)	No. blastocyst (%)	Cell number of blastocyst
Control	198	124 (62.6)	38 (19.2)^a^	105.0±8
None-sense siRNA	191	118 (62.0)	31 (16.2)^a^	90.0±3
*SGO1* siRNA	184	123 (66.7)	17 (9.4)^b^	65.0±5

Within a column, values with different superscripts differ significantly, p<0.05.

### Depletion of SGO1 in Bovine Embryonic Fibroblast Cells Induces Mitotic Arrest

**Figure 6:**
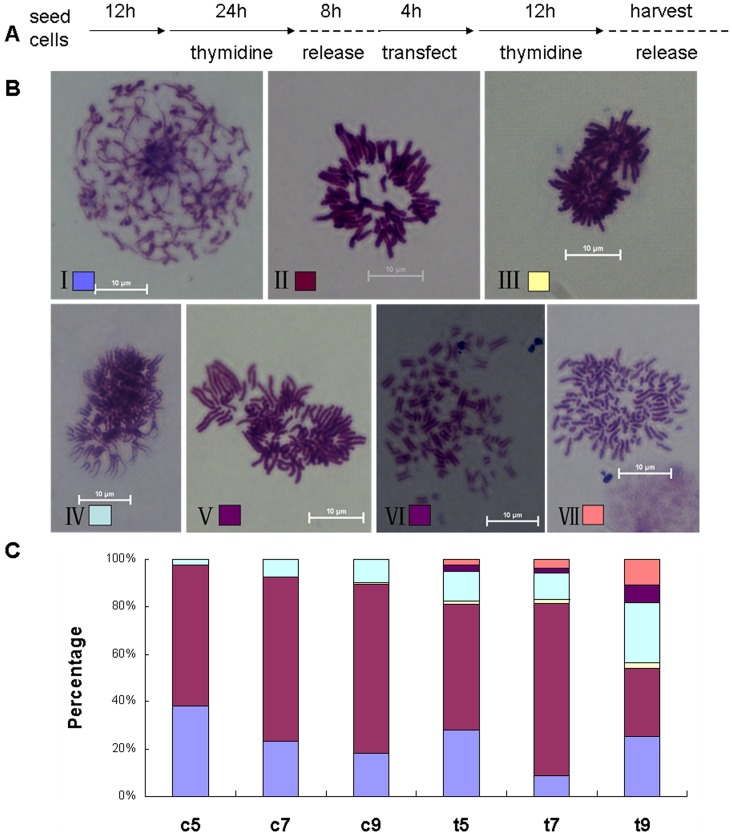
*SGO1* depletion in bovine mitosis caused various types of mitotic arrestment. A) Schematic overview of experimental process. B) Representative images of seven kinds of mitotic patterns. I: prophase. II: metaphase and metaphase-like. III: anaphase. IV: Early separation of sister chromatid. Some or all arm cohesion of the chromosomes seems to be lost and displays a “V” appearance. V: At this stage, centromeric cohesion appears nonexistent. Some or all sister chromatids are still aligned in pairs. VI: Chromosomal composition appears as in V, but is hyper condensed state. VII: Sister chromatids are completely separated and hyper condensed, individual chromatids are scattered disorderly. Bar = 10 µm; original magnification×1000. C) Percentages of each kind of mitotic cells showed. c5, c7, c9 stand for non-sense siRNA treated cells harvest at 5 h, 7 h and 9 h after release 2, t5, t7, t9 stand for *SGO1* specific siRNA treated cells harvest at 5 h, 7 h and 9 h after siRNA treatment.

To further verify whether SGO1 had similar effects on somatic cells as observed in oocytes, bovine embryonic fibroblast cells were synchronized and transfected with *SGO1* siRNAs as shown in Fig. 6A. Samples were collected at 5, 7 and 9 h post treatment. After *SGO1* was depleted, chromosomal alignment and chromosome spread appearance were classified into seven different pattern types (Fig. 6B). Pattern 1 occurred during prophase when the thread-like chromatin condensed (Fig. 6B-I). Pattern 2 displays a metaphase-like chromosomal alignment with sister chromatid pairs orderly aligned at the equatorial plate (Fig. 6B-II). Pattern 3 shows cells at anaphase or telophase with sister chromatids fully separated into two clusters (Fig. 6B-III). In pattern 4, all chromosomes condense into shortened structures. Nearly all of the cohesion between sister chromatid arms has disappeared in this pattern. Most of the chromatid pairs are connected at the centromere and displayed a prolonged “V” shape structure (Fig. 6B-IV). As observed in pattern 5, centromeric cohesion has totally dissipated and arm cohesion has disappeared in some or all of the chromosomes. Sister chromatids are still aligned in pairs although somewhat further separated from each other. Chromosomes observed in this pattern were not regularly aligned at the equatorial plate, but rather scattered about (Fig. 6B-V). In pattern 6, chromosomal alignment was similar to pattern 5, but the chromosomes were hyper condensed and scattered out more random fashion (Fig. 6B-VI). In pattern 7, sister chromatids were completely separated, hyper condensed and very scattered. Single chromatids were shorter and had the appearance of a curled rod-like structure. Chromatids were scattered in a disorderly fashion with no indication that they were being pulled to opposite spindle poles and cell division progressed. The cohesion between chromosomal arms and at the centromeres was non existent (Fig. 6B-VII). Patterns 5, 6 and 7 were observed only in *SGO1*-depleted cells.

In the non-sense RNAi group, 40% of the cells were at prophase 5 h post-treatment, decreasing to 20% at 9 h (Fig.6B-I). More than 50% of the cells remained in metaphase until 9 h post treatment (Fig.6B-II). Cells progressed to anaphase 9 h post-release (Fig. 6BIII). Cells with “V” shape chromatids gradually increased (Fig. 6B-IV). Mitotic patterns 5, 6 and 7 were not observed in the non-sense treated cells.

In the *SGO1*-depleted group, more cells were observed lacking chromosomal arm cohesion and with prolonged “V” shape chromosomes. This was especially observable at 9 h where this condition occurred in 25.3% of the cells (Fig. 6BIV). These anomalies were significantly higher than that in the non-sense depleted cells. It appears that *SGO1* depletion causes a premature dissociation of chromosomal arm cohesions. Cells in anaphase at 9 h in non-sense treated cells emerged at 5 h in *SGO1*-depleted cells and increased over time (Fig.6BIII), although only a small part of mitotic cells will normally entry anaphase precociously. *SGO1* depletion induces the premature dissociation of cohesion along the chromosomal arms and at the centromere. Mitotic patterns 5 - 7 were only observed in *SGO1-* depleted cells and their associated chromosome anomalies increased significantly (Fig. 6BV-VII, Fig. 6C). Unattached chromatids were quite condensed and dispersed individually. Cells with these characteristics failed to enter anaphase and became mitotically arrested, which oftentimes formed polyploids. The functions or roles of SGO1 in bovine embryonic fibroblasts were very similar to what was observed in the oocyte/embryo studies.

## Discussion

Shugoshin proteins have been studied mostly in yeast, fruit flies, *Xenopus*, Hela cells and plants. Studies on their role in the regulation of meiosis have mainly come from studies in yeast [8], [11], [12] and invertebrates [7]. SGO in a mammalian system has only been reported in the mouse [23]–[25], [41]. To the best of our knowledge this is the first study in the bovine where we report on the function of SGO1 during bovine meiosis and mitosis and the effects of *SGO1* knockdown. An exogenously delivered *SGO1* with different concentrations were microinjected into oocytes at the GV stage to examine the localization of SGO1 during bovine oocyte meiosis and its effects on chromosomal separation upon over-expression. RNAi of *SGO1* was conducted to examine the effect of SGO1 on meiotic processes and subsequent embryonic development. For comparative purposes, the role of SGO1 was also studied during mitosis.

Exogenous mouse SGO1 was used to localize and observe its action. SGO1 was observed along the entire chromosomal arm and concentrated at the centromeres until metaphase I. At anaphase I, SGO1 staining on the chromosomal arms decreased while centromere staining remained traceable until metaphase II. SGO1 could not be detected in the polar bodies. When sister chromatids separated, none of the characteristic SGO1 signals were observable [41]. Endogenous mouse SGO1 localized at the centromere during meiosis I and meiosis II, and was maintained in that state until early anaphase II [24]. Sgo1 in *Xenopus* extracts were extensively distributed during interphase and accumulated rapidly at the centromere once the cell entered into mitosis [42]. The staining intensity of Sgo1 in *Xenopus* cells at prophase and prometaphase weakened at metaphase and disappeared completely at anaphase [6]. The only homolog of SGO reported in budding yeast colocalized with the spindle pole from G1 to the S phase and was distributed over the entire nucleus at metaphase, and then disappated at anaphase in mitotic cells. Budding yeast *SGO1* was expressed higher in meiosis, especially during meiosis I than in mitosis. Sgo1 was first detected at the kinetochore beginning at pachytene and then maintained through to metaphase II [11]. Bovine SGO1 was first observed in pre-MI chromosomes and was continuously maintained to MII, spread all over the nucleus. The SGO1-MYC signal that was observed at pre-metaphase of meiosis II was not detectable in the polar body, which is a pattern similar to what has been reported in the mouse SGO1 [41]. The force emanating between the bipolar spindle attachment and the kinetochore aligns the chromatids on the metaphase plate [43]. The loss of sister chromatid cohesion and the resultant dynamic variation in chromosome behavior is closely tied to SGO1. Studies on human cells [4], [44], *Xenopus* extracts [6] and budding yeast [45] have supports the role that SGO1 regulates the connection of the kinetochore and the microtubules. Such spindle architecture is hardly to see in the polar body. We hypothesize, based upon the observations from this study, that SGO1 in the bovine is associated with the recruitment of microtubules at the kinetochore as has been reported to occur in other species.

It is generally accepted that during oocyte maturation, there is no transcription activity. Our quantative analysis results showed that bovine *SGO1* mRNA level was decreased at germinal vesicle. After GVBD, the amount of *SGO1* mRNA increased gradually until metaphase II stage and showed a dramatic increase 16 h post maturing. We speculate that *SGO1* transcription is re-activated once GVBD during bovine oocyte *in vitro* maturation under our condition at least. Whether it’s just exception for specific genes such as factors involved in chromosomal separation or not needs to be further investigated. The fact that *SGO1* expressed more in meiosis II than in meiosis I indicated its potential roles preferred to chromatid separation rather than homologous chromosomal separation process. This hypothesis was verified via the over expression and RNAi of *SGO1.*Unlike what has been reported in mouse oocytes [41], homologous chromosomal separation in the bovine was not affected when *SGO1* was over-expressed. None-the-less, however, separated chromosomes could not be pulled apart and transported to opposite spindle poles. Clift et al.(2009) reported that over expression of *SGO1* in the budding yeast causes mitotic arrestment [46]. Over expression of *SGO1* in the bovine oocyte resulted in meiotic arrestment and a decrease in maturation rate. Therefore, it appears that bovine SGO1 is involved in the movement of chromosomes towards the poles. Excessive amounts of SGO1 somehow antagonize the pull of the microtubules preventing the formation of haploid gametes. SGO1 may simply be involved in tension sensing and setting at the centromere during mitosis as has been reported in Hela cells [4], *Xenopus* extracts [6] and budding yeast [45]. We hypothesize that over-expression of SGO1 causes aberrant chromosome capture of microtubules and abnormal tension-sensing at the kinetochore, which then accounts for the irregular and disordered chromosomal alignment. It appears that SGO1 in the bovine model is associated with chromosomal polarization during meiotic maturation in *SGO1*-depleted oocytes. *SGO1* depletion causes oocytes to arrest during meiosis as the chromosomes fail to migrate to opposite spindle poles, thus thwarting normal separation and inducing premature chromatid separation. The observations also suggest that, as in other models [8], [11], [12], [14], [41], that bovine SGO1 is responsible for centromere cohesion of sister chromatids during meiosis. The involvement of centromere cohesion was also confirmed in mitosis.

The mitotic processes in bovine fibroblast cells were analyzed in similar detail to the meiotic processes that occurred in the bovine oocyte/embryo. Seven unique mitotic configuration patterns were observed during *SGO1* depletion and classified into distinctive groups (shown in Fig. 6B). Patterns 5, 6 and 7 were observed only in the *SGO1*-depleted cells. Depletion of *SGO1* caused dissociation of cohesion at the centromere on some or all chromosomal arms. This resulted in mitotic arrestment. Paired sister chromatids were pulled further apart from each other to the point they eventually formed hyper condensed and individually recognized chromosomes. This was probably the primary cause leading to the formation of polyploid cells. The effects on mitosis seemed to be similar to what we observed during meiosis. SGO1 protects centromeric cohesion, which in consistent with similar reports in *Xenopu*s cells [6] and Hela cells [6], [17].

In conclusion, bovine SGO1 is associated with chromosomal polarization during meiosis and involves centromeric cohesion of sister chromatids during both meiosis and mitosis. Depletion of *SGO1* will result in the arrestment of meiotic and mitotic cell division. SGO1 was essential for the faithful chromosomal separation.

## References

[1] Petronczki M, Siomos MF, Nasmyth K (2003) Un menage a quatre: the molecular biology of chromosome segregation in meiosis. Cell 112: 423–440. 12600308 10.1016/s0092-8674(03)00083-7

[2] Watanabe Y (2005) Shugoshin: guardian spirit at the centromere. Curr Opin Cell Biol 17: 590–595. 16229998 10.1016/j.ceb.2005.10.003

[3] Gutié rrez-Caballero C, Cebollero LR, Pendá s AM (2012) Shugoshins: from protectors of cohesion to versatile adaptors at the centromere. Trends Genet 28(7): 351–360. 22542109 10.1016/j.tig.2012.03.003

[4] Pouwels J, Kukkonen AM, Lan WJ, Daum JR, Gorbsky GJ, et al (2007) Shugoshin 1 plays a central role in kinetochore assembly and is required for kinetochore targeting of Plk1. Cell Cycle 6: 1579–1585. 17617734 10.4161/cc.6.13.4442

[5] Tang Z, Sun Y, Harley SE, Zou H, Yu H (2004) Human Bub1 protects centromeric sister-chromatid cohesion through Shugoshin during mitosis. PNAS 101: 18012–18017. 15604152 10.1073/pnas.0408600102 PMC539817

[6] Salic A, Waters JC, Mitchison TJ (2004) Vertebrate Shugoshin links sister centromere cohesion and kinetochore microtubule stability in mitosis. Cell 118: 567–578. 15339662 10.1016/j.cell.2004.08.016

[7] Kerrebrock AW, Moore DP, Wu JS, Orr-Weaver TL (1995) Mei-S332, a Drosophila protein required for sister-chromatid cohesion,can localize to meiotic centromere regions. Cell 83: 247–256. 7585942 10.1016/0092-8674(95)90166-3

[8] Kitajima TS, Kawashima SA, Watanabe Y (2004) The conserved kinetochore protein shugoshin protects centromeric cohesion during meiosis. Nature 427: 510–517. 14730319 10.1038/nature02312

[9] Hamant O, Golubovskaya I, Meeley R, Fiume E, Timofejeva L, et al (2005) A Rec8-dependent plant Shugoshin is required for maintenance of centromeric cohesion during meiosis and has no mitotic functions. Curr Biol 15: 948–954. 15916952 10.1016/j.cub.2005.04.049

[10] Wang M, Tang D, Wang K, Shen Y, Qin B, et al (2011) OsSGO1 maintains synaptonemal complex stabilization in addition to protecting centromeric cohesion during rice meiosis. Plant J 67: 583–594. 21615569 10.1111/j.1365-313X.2011.04615.x

[11] Katis VL, Galova M, Rabitsch KP, Gregan J, Nasmyth K (2004) Maintenance of cohesin at centromeres after meiosis I in budding yeast requires a kinetochore-associated protein related to MEI-S332. Curr Biol 14: 560–572. 15062096 10.1016/j.cub.2004.03.001

[12] Rabitsch KP, Gregan J, Schleiffer A, Javerzat JP, Eisenhaber F, et al (2004) Two fission yeast homologs of Drosophila Mei-S332 are required for chromosome separation during meiosis I and II. Curr Biol 14: 287–301. 14972679 10.1016/j.cub.2004.01.051

[13] Dudas A, Ahmad S, Gregan J (2011) Sgo1 is required for co-segregation of sister chromatids during achiasmate meiosis I. Cell Cycle. 10(6): 951–955. 10.4161/cc.10.6.15032 PMC3100875 21330786

[14] Kerrebrock AW, Miyazaki WY, Birnbyt D, Orr-Weave TL (1992) The Drosophila mei-S332 gene promotes sister-chromatid cohesion in meiosis following kinetochore differentiation. Genetics 130: 827–841. 1582560 10.1093/genetics/130.4.827 PMC1204932

[15] Shintomi K, Hirano T (2009) Releasing cohesin from chromosome arms in early mitosis: opposing actions of Wapl -Pds5 and Sgo1. Genes Dev 23: 2224–2236. 19696148 10.1101/gad.1844309 PMC2751989

[16] Tang Z, Shu H, Qi W, Mahmood NA, Mumby MC, et al (2006) PP2A is required for centromeric localization of Sgo1 and proper chromosome separation. Dev Cell 10: 575–585. 16580887 10.1016/j.devcel.2006.03.010

[17] McGuinness BE, Hirota T, Kudo NR, Peters JM, Nasmyth K (2005) Shugoshin prevents dissociation of cohesin from centromeres during mitosis in vertebrate cells. PLos Biol 3: e86. 15737064 10.1371/journal.pbio.0030086 PMC1054882

[18] Kawashima SA, Tsukahara T, Langegger M, Hauf S, Kitajima TS, et al (2007) Shugoshin enables tension-generating attachment of kinetochores by loading Aurora to centromeres. Genes Dev 21(4): 420–435. 17322402 10.1101/gad.1497307 PMC1804331

[19] Vanoosthuyse V, Prykhozhij S, Hardwick KG (2007) Shugoshin 2 regulates localization of the chromosomal passenger proteins in fission yeast mitosis. Mol Biol Cell 18: 1657–1669. 17301288 10.1091/mbc.E06-10-0890 PMC1855032

[20] Kitajima TS, Sakuno T, Ishiguro K, Iemura S, Natsume T, et al (2006) Shugoshin collaborates with protein phosphatase 2A to protect cohesin. Nature 441: 46–52. 16541025 10.1038/nature04663

[21] Huang HM, Feng J, Famulski J, Rattner JB, Liu ST, et al (2007) Tripin/hSgo2 recruits MCAK to the inner centromere to correct defective kinetochore attachments. J Cell Biol 177: 413–424. 17485487 10.1083/jcb.200701122 PMC2064832

[22] Kitajima TS, Hauf S, Ohsugi M, Yamamoto T, Watanabe Y (2005) Human Bub1 defines the persistent cohesion site along the mitotic chromosome by affecting Shugoshin localization. Curr Biol 15: 353–359. 15723797 10.1016/j.cub.2004.12.044

[23] Llano E, Gómez R, Gutiérrez-Caballero C, Herrán Y, Sánchez-Martín M, et al (2008) Shugoshin-2 is essential for the completion of meiosis but not for mitotic cell division in mice. Genes Dev 22: 2400–2413. 18765791 10.1101/gad.475308 PMC2532928

[24] Lee J, Kitajima TS, Tanno Y, Yoshida K, Morita T, et al (2008) Unified mode of centromeric protection by shugoshin in mammalian oocytes and somatic cells. Nat Cell Biol 10: 42–52. 18084284 10.1038/ncb1667

[25] Mailhes JB, Hilliard C, Fuseler JW, London SN (2003) Okadaic acid, an inhibitor of protein phosphatase 1 and 2A, induces premature separation of sister chromatids during meiosis I and aneuploidy in mouse oocytes in vitro. Chromosome Res 11: 619–631. 14516070 10.1023/a:1024909119593

[26] Liu Y, Li GP, White KL, Rickords LF, Sessions BR, et al (2007) Nicotine alters bovine oocyte meiosis and affects subsequent embryonic development. Mol Reprod Dev 74: 1473–1482. 17440977 10.1002/mrd.20717

[27] Liu Y, Li GP, Sessions BR, Rickords LF, White KL, et al (2008) Nicotine induces multinuclear formation and causes aberrant embryonic development in bovine. Mol Reprod Dev 75: 801–809. 18157849 10.1002/mrd.20774

[28] Rajagopalan H, Lengauer C (2004) Aneuploidy and cancer. Nature 432: 338–341. 15549096 10.1038/nature03099

[29] Bharadwaj R, Yu H (2004) The spindle checkpoint, aneuploidy, and cancer. Oncogene 23: 2016–2027. 15021889 10.1038/sj.onc.1207374

[30] Malmanche N, Maia A, Sunkel CE (2006) The spindle assembly checkpoint: preventing chromosome mis-segregation during mitosis and meiosis. FEBS Lett 580: 2888–2895. 16631173 10.1016/j.febslet.2006.03.081

[31] Weaver BA, Cleveland DW (2006) Does aneuploidy cause cancer? Curr Opin Cell Biol. 18 658–667. 10.1016/j.ceb.2006.10.002 17046232

[32] Dai W (2009) Suppression of genomic instabilities caused by chromosome mis-segregation: A perspective from studying BubR1 and Sgo1. Formos Med Assoc 108(12): 904–911. 10.1016/S0929-6646(10)60002-2 PMC3763510 20040454

[33] Yamada HY, Yao Y, Wang X, Zhang Y, HuangY, et al (2012) Haploinsufficiency of *SGO1* results in deregulated centrosome dynamics, enhanced chromosomal instability and colon tumorigenesis.Cell cycle. 11(3): 479–488. 10.4161/cc.11.3.18994 PMC3315092 22262168

[34] Iwaizumi M, Shinmura K, Mori H, Yamada H, Suzuki M, et al (2009) Human Sgo1 downregulation leads to chromosomal instability in colorectal cancer. Gut 58: 249–260. 18635744 10.1136/gut.2008.149468

[35] Scanlan MJ, Gout I, Gordon CM, Williamson B, Stockert E, et al (2001) Humoral immunity to human breast cancer: antigen definition and quantitative analysis of mRNA expression. Cancer Immun 1: 4. 12747765

[36] Yang YL, Wang X, Dai W (2006) Human Sgo1 is an excellent target for induction of apoptosis of transformed cells. Cell Cycle 5 (8): 896–901. 10.4161/cc.5.8.2691 16628005

[37] Li GP, Bunch TD, White KL, Aston KI, Meerdo LN, et al (2004) Development, chromosomal composition and cell allocation of bovine cloned blastocyst derived from chemically assisted enucleation and cultured in conditioned media. Mol Reprod Dev 68: 189–197. 15095340 10.1002/mrd.20071

[38] Livak KJ, Schmittgen TD (2001) Analysis of relative gene expression data using real-time quantitative PCR and the 2(-Delta Delta C (T)) Method. Methods 25: 402–408. 11846609 10.1006/meth.2001.1262

[39] Kiburz BM, Amon A, Marston AL (2008) Shugoshin promotes sister kinetochore biorientation in Saccharomyces cerevisiae. Mol Biol Cell 19: 1199–1209. 18094053 10.1091/mbc.E07-06-0584 PMC2262988

[40] Li GP, Liu Y, Bunch TD, White KL, Aston KI (2005) Asymmetric division of spindle microtubules and microfilaments during bovine meiosis from metaphase I to metaphase III. Mol Reprod Dev 71: 220–226. 15791589 10.1002/mrd.20255

[41] Yin S, Ai JS, Shi LH, Wei L, Yuan J, et al (2008) Shugoshin1 may play important roles in separation of homologous chromosomes and sister chromatids during mouse oocyte meiosis. PLos One 3: e3516. 18949044 10.1371/journal.pone.0003516 PMC2567865

[42] Rivera T, Losada A (2009) Shugoshin regulates cohesion by driving relocalization of PP2A in Xenopus extracts. Chromosoma 118: 223–233. 18987869 10.1007/s00412-008-0190-4

[43] Nicklas RB (1988) The forces that move chromosomes in mitosis. Annu. Rev. Biophys Biophys Chem 17: 431–449. 10.1146/annurev.bb.17.060188.002243 3293594

[44] Suzuk H, Akiyama N, Tsuji M, Ohashi T, Saito S, et al (2006) Human Shugoshin mediates kinetochore-driven formation of kinetochore microtubules. Cell Cycle 5: 1094–1101. 16687935 10.4161/cc.5.10.2747

[45] Indjeian VB, Stern BM, Murray AW (2005) The centromeric protein Sgo1 is required to sense lack of tension on mitotic chromosomes. Science 307: 130–133. 15637284 10.1126/science.1101366

[46] Clift D, Bizzari F, Marston AL (2009) Shugoshin prevents cohesin cleavage by PP2A^Cdc55^-dependent inhibition of separase. Genes Dev 23: 766–780. 19299562 10.1101/gad.507509 PMC2661608

